# Study on the Hydrophobic Modification Mechanism of Stearic Acid on the Surface of Coal Gasification Fly Ash

**DOI:** 10.3390/molecules29174071

**Published:** 2024-08-28

**Authors:** Jian Yang, Longjiang Li, Wenyuan Wang

**Affiliations:** 1 Mining College, Guizhou University, Guiyang 550025, China; 18230701027@163.com (J.Y.); 18744774725@163.com (W.W.); 2 National & Local Joint Laboratory of Engineering for Effective Utilization of Regional Mineral Resources from Karst Areas, Guiyang 550025, China; 3 Guizhou Key Laboratory of Comprehensive Utilization of Nonmetallic Mineral Resources, Guiyang 550025, China

**Keywords:** hydrophobic structure, coulomb repulsion, active site, surface modified, fly ash

## Abstract

In this study, the hydrophobic modification of coal gasification fly ash (FA) was investigated given the adverse effects of surface hydrophilic structures on the material field. The surface of FA was modified using stearic acid (SA), which successfully altered its hydrophilic structure. When the contact angle of S-FA increased from 23.4° to 127.2°, the activation index increased from 0 to 0.98, the oil absorption decreased from 0.564 g/g to 0.510 g/g, and the BET-specific surface area decreased from 13.973 m^2^/g to 3.218 m^2^/g. The failure temperature of SA on the surface of S-FA was 210 °C. The adsorption mechanism of FA was analyzed using density functional theory (DFT) and molecular dynamics (MD). The adsorption of water molecules by FA involved both chemical and physical adsorption, with active adsorption sites for Al, Fe, and Si. The adsorbed water molecules on the surface of FA formed hydrogen bonds with a bond length of 1.5–2.5 Å, leading to agglomeration. In addition, the long alkyl chain in SA mainly relied on the central carbon atom in the (-CH_3_) structure to obtain electrons in different directions from the H atoms in space, increasing the Coulomb repulsion with the O atoms in the water molecule and thereby achieving the hydrophobic effect. In the temperature range of 298 K to 358 K, the combination of FA and SA became stronger as the temperature increased.

## 1. Introduction

Coal gasification fly ash (FA) is a solid gasification byproduct generated by the reaction of unreacted coal particles or coal particles with gasification agents in a gasifier, serving as a kind of fine particle captured after gas removal, condensation, and agglomeration. If a large amount of FA is not treated, it produces dust [1], pollutes the atmosphere, and causes other environmental problems. FA contains a variety of heavy metal elements, and large-scale storage can cause groundwater pollution [2].

The chemical composition and crystal phase composition of FA are very similar to those of fly ash, and scholars generally do not distinguish between them. At present, the comprehensive utilization of FA includes catalysis [3], valuable metal recovery [4,5], agriculture, adsorption materials [6,7,8], and building materials [9,10]. However, the production of FA is also increasing with the large-scale construction of coal chemical plants. Many scholars have carried out modification research on FA to achieve large-scale utilization. Through the mechanical grinding method [11], acid‒base modification method [12,13], high-temperature treatment method [14,15], surface modification method [16], etc., the porosity of FA increases, the specific surface area increases, and the adsorption capacity of FA further improves. Among them, the surface modification method is the best and the most commonly used method for changing the surface properties of FA, and the effect after modification often meets the expectations of the tester.

FA has the advantages of porosity and a large specific surface area, which results in good adsorption performance, but its water absorption and hydrophilicity limit its comprehensive utilization. In the process of preparing concrete using FA, porous water absorption [17] consumes too much free water in the system, which adversely affects the fluidity of the system and results in a retarding phenomenon [18,19,20] and the poor strength performance of the concrete. Lei Qu et al. [21] modified the surface of FA with stearic acid (SA), which alleviated the early poor resistance to external water seepage and corrosion of FA cement mortar and promoted the hydration reaction of cement, making the internal structure of mortar more compact [22]. FA shows great potential in removing phenolic compounds from wastewater [23]. By regulating the surface hydrophobicity and nonpolarity of FA, the cost-effectiveness of easy batch production and continuous operation, reuse, and regeneration, and no sludge generation can be obtained. FA-based zeolite [24] is nonflammable, can be calcined and regenerated, and has controllable surface hydrophobicity, which gives it the ability to selectively adsorb volatile organic compounds, effectively alleviating environmental problems such as photochemical pollution and greenhouse gases. The surface hydrophobicity and nonpolarity regulation of FA effectively broadens the comprehensive utilization of FA, partially replacing cement and lime in the preparation of autoclaved aerated bricks, reducing the amount of other raw materials, and solving the problem of wastewater treatment containing phenols. However, the structural properties of ash samples produced using different production processes differ greatly. There is no research focusing on the hydrophilic water absorption performance of FA before and after surface modification. Moreover, it is extremely difficult to analyze the effects of surface modification on the water absorption structure of FA through experiments alone. Therefore, the hydrophobic mechanisms of FA surface modification and the interaction mechanism with water need to be further explored.

In this study, SA was coated on the surface of FA via dry modification. The structure of the FA before and after modification was characterized using modern instruments. Combined with computational chemistry methods, the hydrophilicity and hydrophobicity of the FA and the hydrophobic mechanism of the modified FA surface were further investigated from a microscopic point of view, which laid a solid theoretical foundation for the use of hydrophobic FA as a building material and an adsorption material.

## 2. Results and Discussion

### 2.1. FA Characteristics

**Figure 1:**
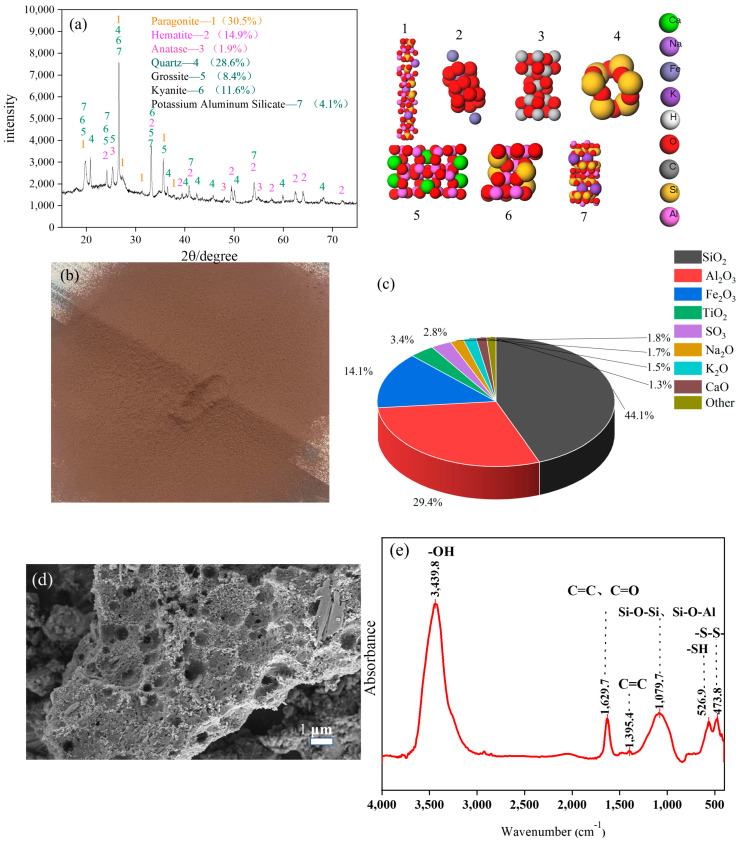
Characteristics of the FA: (**a**) XRD pattern; (**b**) powder diagram; (**c**) XRF result; (**d**) SEM image of; (**e**) FT-IR spectrum.

As shown in Figure 1a, FA is composed of sodium mica, quartz, hematite, and anatase.Figure 1b shows the powder diagram of coal gasification FA, with a dark red color. The content of hematite is as high as 14.9%, which is the main reason for the dark red color of FA. Sodium mica, quartz, and hematite have good hydrophilicity, endowing the surface of FA with good wettability. Figure 1c shows the XRF spectrum of FA as containing large amounts of SiO_2_, Al_2_O_3_, and Fe_2_O_3_. The SiO_2_ content is as high as 44.1%, and a large amount of silicon gives FA good chemical stability, providing the potential for use as a high-temperature and corrosion-resistant material. Figure 1d shows the SEM image of FA. There are many micropores, mesopores, and macropores on the surface of FA, which led to the strong water absorption of FA. In addition, FA is flaky without a microspherical structure, which is different from the morphology of fly ash. The spherical structure of fly ash improves the fluidity of the slurry, while FA with a flaky structure adversely affects the fluidity of the slurry system. Figure 1e shows the FT-IR spectrum of FA. The absorption peak at 3439.8 cm^−1^ was attributed to alcohol and phenolic-OH (hydroxyl) on the surface of FA, belonging to the common characteristic groups of coal gasification solid waste under different gasification processes. The absorption peak at 1629.7 cm^−1^ was assigned to the stretching vibration of olefin C=C and carbonyl C=O, the absorption peak at 1395.4 cm^−1^ was caused by the C=C stretching vibration of the aromatic ring, the absorption peak at 1079.7 cm^−1^ was related to Si-O-Si and Si-O-Al stretching vibrations, the absorption peak at 562.9 cm^−1^ was attributed to the SH stretching vibration, and the absorption peak at 473.8 cm^−1^ was referenced as the S‒S stretching vibration peak. These oxygen-containing functional groups, such as OH and carbonyl C=O, Si-O-Si, and other groups, facilitated hydrogen bond adsorption with water molecules, thereby further improving the hydrophilicity of the FA surface. In addition, the absorption peak of the nitrogen-oxygen thiol (-SH) group indicated the presence of some functional groups containing nitrogen and sulfur in FA.

### 2.2. FA Modification Characteristics

**Figure 2:**
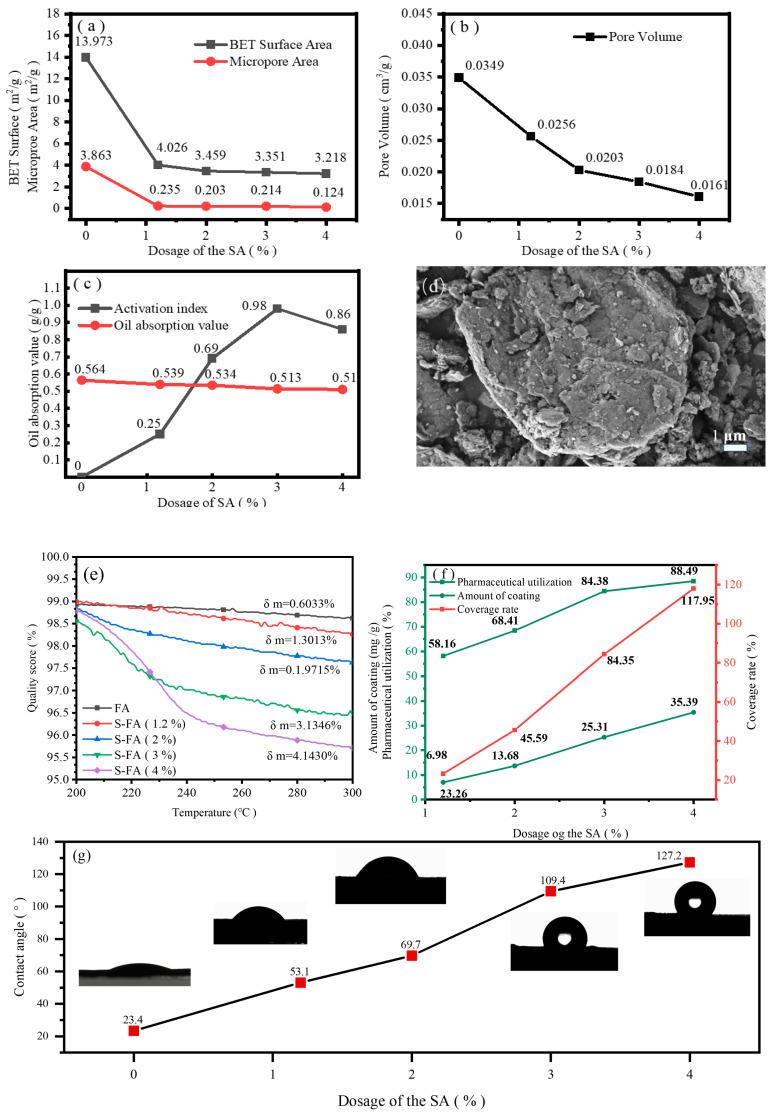
(**a**) Bet surface area and micropore area of S-FA; (**b**) pore volume of S-FA; (**c**) activation index and oil absorption value of S-FA; (**d**) SEM image of S-FA; (**e**) thermogravimetric curve of S-FA; (**f**) coating rate and coating rate of SA; (**g**) contact angle of S-FA (The black graphic is a photo taken by the contact angle measuring instrument).

Figure 2a shows the specific surface area and micropore area of S-FA. With increasing amounts of SA from 0 to 4%, the BET-specific surface area of S-FA decreased from 13.973 m^2^/g to 3.218 m^2^/g, which is a decrease in microporous specific surface area from 3.863 m^2^/g to 0.124 m^2^/g (a 97% decrease). Figure 2b shows the pore volume change curve of S-FA. The pore volume decreased from 0.0349 cm^3^/g to 0.0161 cm^3^/g, which indicates that SA molecules were adsorbed into the pores of FA, thus lowering the adsorption capacity of FA [25]. Figure 2d shows the SEM image of S-FA. Compared with Figure 1d, there were almost no micropores on the surface of S-FA, with small particles adhering to the surface of large particles, which suggests the decrease in the specific surface area of FA caused by the pore-blocking effect and the adhesion between particles of SA.

#### 2.2.1. Activation Index and Oil Absorption Value

As shown in Figure 2c, the surface of unmodified FA was in a polar state, and the activation index was 0. All the ash samples were naturally settled in water, and the activation index reached 0.98 when SA (3%) was used to modify the FA. At this time, the surface of the FA particles was coated with a layer of SA. With an increase in the SA dose to 4%, the activation index decreased to 0.86, which indicated that excessive SA formed a weak physical adsorption interface and affected the uniformity of the SA monolayer on the surface of the FA particles. With an increase in the amount of SA from 0 to 4%, the oil absorption value of FA decreased from 0.564 g/g to 0.510 g/g, which demonstrated that the use of S-FA as an inorganic filler had a beneficial effect on the cost and processing performance of the resulting molding compounds. Combined with Figure 2a, when the amount of SA increased from 1.2% to 3%, the micropore-specific surface area of FA decreased by only 0.021 m^2^/g, but the activation index increased from 0.25 to 98%. When the amount of SA is 1.2%, SA almost completely blocked FA. Priority to achieving the plugging effect is given to starting the surface coating outside the hole. Therefore, the main factor affecting the amount of SA is the pore adsorption of FA. Less expensive and better plugging agents before surface modification can effectively reduce the amounts of modified agents.

#### 2.2.2. Thermogravimetric Curve

As shown in Figure 2e, the weight changes of different S-FAs varied significantly in the range of 200 °C to 300 °C. The accelerated decomposition of SA occurred at 210 °C, and S-FA began to fail at the temperature. Above 300 °C, SA completely decomposed and volatilized from the surface of FA. Through dynamic regulation in the temperature range of 200–300 °C, the volatile decomposition amount of SA can be controlled, thereby dynamically adjusting the surface hydrophobicity, oil absorption value, and nonpolarity of S-FA. As shown in Figure 2f, the utilization rate of SA increased from 58.16% to 84.38% when the dose of SA increased from 1.2% to 3%, which indicates that there was a synergistic effect between SA molecules when SA was coated on the surface of FA. The coating rate reached 84.35% when the amount of SA was 3%. According to the theoretical calculations, the coating rate should reach 100%, and the amount of SA should be 3.5%. When the amount of SA was 4%, the theoretically calculated coating rate reached 117.95%, which suggests that the amount of SA exceeded the optimal amount of SA monolayer formed on the surface of FA. Combined with Figure 2c, when the activation index was the highest, the SA monolayer coating was completed on the surface of FA. When the amount of SA exceeded the amount of monolayer coating, multilayer SA molecules were formed on the surface of FA, and the stability in water was not as good as that of monolayer SA, resulting in an increase in FA and a decrease in the activation index. Contact angle tests of FA and S-FA were carried out to further illustrate the effect of SA modification on the hydrophobicity of the FA surface. As shown in Figure 2g, the contact angle of FA was 23.4°, corresponding to a hydrophilic surface. As the amount of SA increased from 1.2% to 4%, the contact angle of S-F increased from 53.1° to 127.2°, indicating a higher surface hydrophobicity of S-FA. As shown in Section 2.2.1, when the amount of SA was 3%, the activation index reached 0.98, but the contact angle was 109.4°, which did not reach the maximum value, indicating that the size of the contact angle did not completely correspond to the activation index. Figure 2f shows that when the amount of SA was 3%, the coating rate reached 88.35%, which did not reach the maximum value. Increasing the amount of SA can further increase the contact angle, which implies a certain correspondence between the contact angle and the coating rate of SA. The higher the coating rate is, the larger the contact angle, and the better the hydrophobicity.

### 2.3. Research on the Microscopic Mechanism

#### 2.3.1. Model Construction

**Figure 3:**
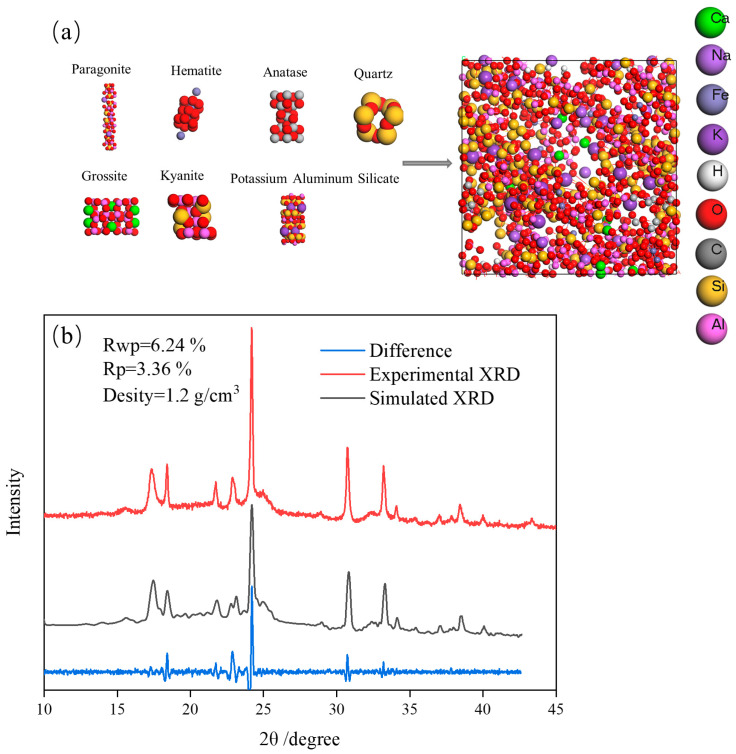

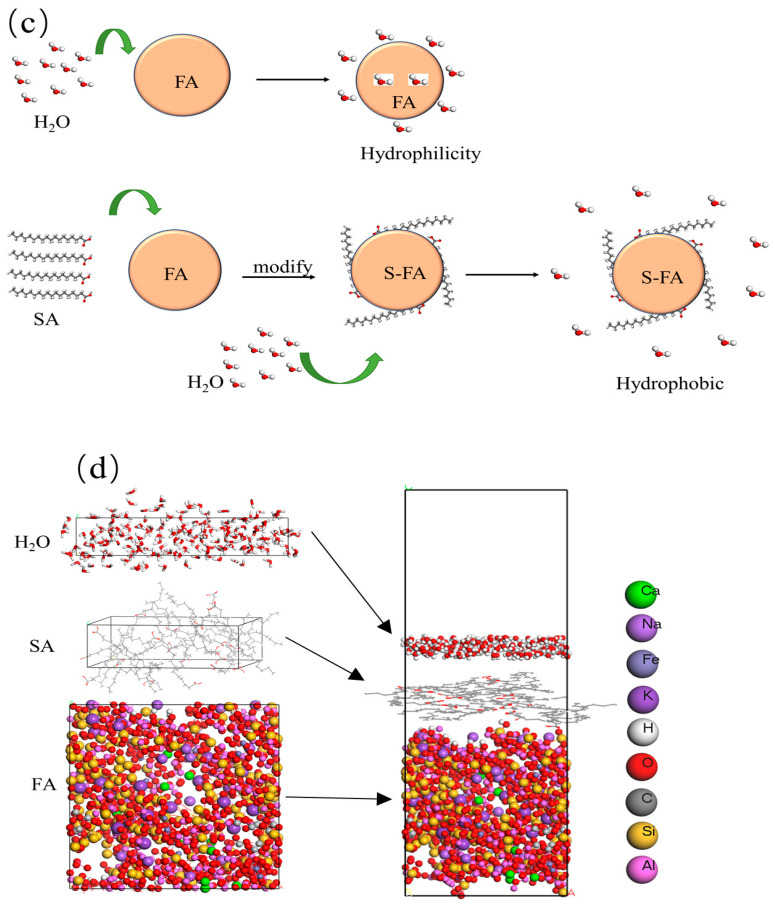
(**a**) initial model of the FA; (**b**) matching degree of the FA model; (**c**) SA modification diagram; (**d**) theoretical model of the S-FA.

Figure 3a shows the initial FA model, the difference between the XRD simulation value of the mixed FA model and the XRD experimental value of FA is shown in Figure 3b. For the amorphous structure, when the weighted pattern residual variance factor Rwp (R-weighted pattern) and the pattern variance factor Rp (R-pattern) were less than 15%, the main XRD peaks of the simulated structure coincide with the main experimental XRD peaks. Models with densities of 1.0 g/cm^3^, 1.2 g/cm^3^, and 1.4 g/cm^3^ were constructed. As shown in Table 1, when the density was 1.2 g/cm^3^, the smallest Rwp and Rp were obtained. As shown in Figure 3b, the XRD pattern of the simulated structure was consistent with the main peak of the experimental XRD pattern, with a relatively flat difference curve, which indicates that the simulated structure and the actual structure have a high degree of similarity. Figure 3c shows a schematic diagram of the surface of S-FA. The surface of FA was modified using SA to repel water molecules. Figure 3d shows the molecular dynamics calculation model of the interaction between S-FA and the water molecular layer, revealing the adsorption and diffusion of water molecules on the surface of S-FA.

#### 2.3.2. Molecular Dynamics Relaxation

**Figure 4:**
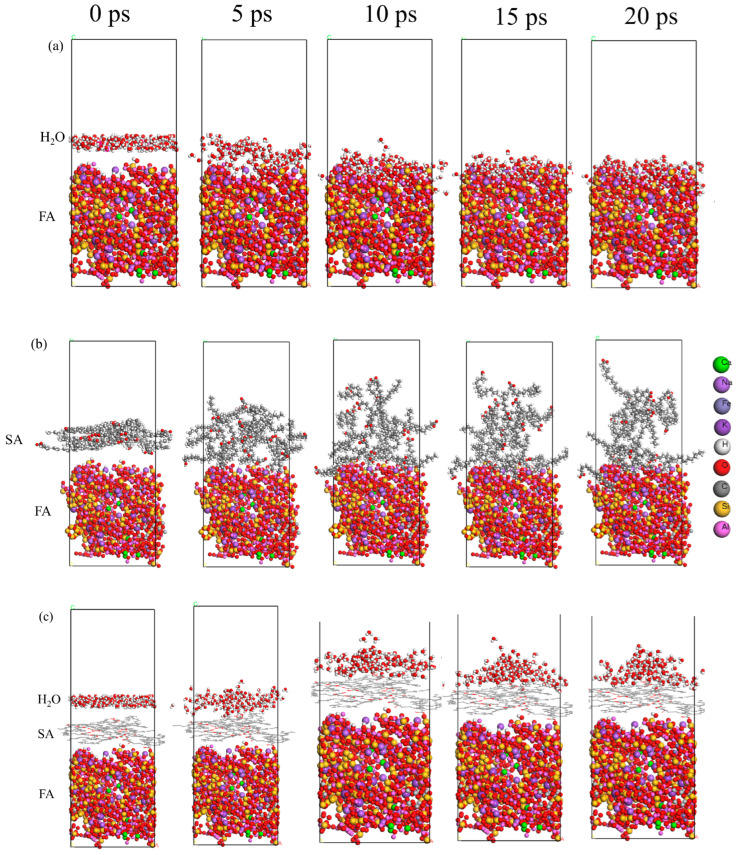
Molecular dynamics relaxation diagram: (**a**) FA and water molecular layer; (**b**) SA and water molecular layer; (**c**) S-FA and water molecular layer.

As shown in Figure 4a, at 298 K, water molecules were adsorbed to the FA surface, began to enter the pores, and finally spread out on the surface, indicating that the FA surface had good wettability. As shown in Figure 4b, SA adsorbed to the surface of FA at 343 K, forming a multilayer organic layer, However, the upper organic layer gradually moved away from the FA surface over time, which was due to the unstable multilayer organic layer formed by excessive SA. As shown in Figure 4c, when an effective SA molecular layer was formed on the surface of FA, the SA molecular layer had a significant repulsive effect on water molecules. Water molecules cannot be tiled on the SA organic surface layer and cross the SA molecular layer, resulting in the inability to adsorb onto the FA surface.

#### 2.3.3. Radial Distribution Function and Coordination Number

**Figure 5:**
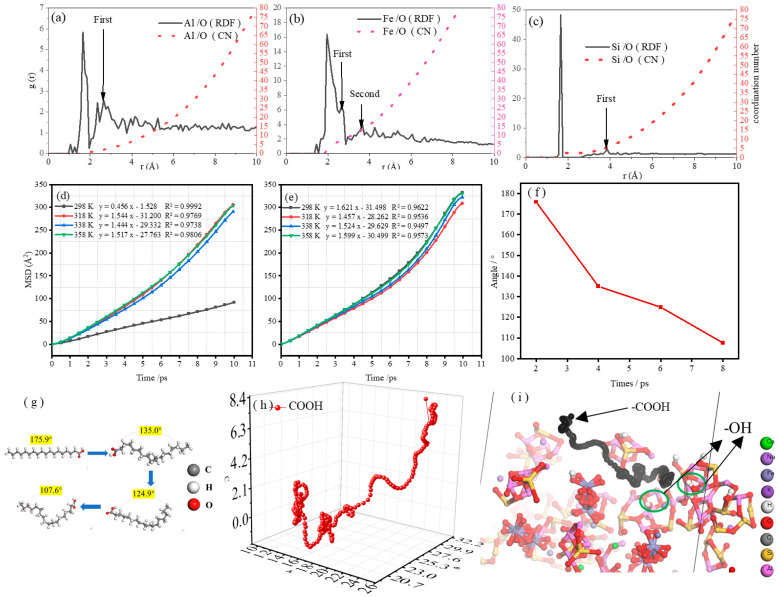
Molecular dynamics: (**a**) radial distribution functions of Al and O; (**b**) radial distribution functions of Fe and O; (**c**) radial distribution functions of Si and O; (**d**) mean square displacement of water molecules on the FA surface; (**e**) mean square displacement of water molecules on the S-FA surface; (**f**,**g**) chain angle of a long chain of SA; (**h**,**i**) the trajectory of the long chain carboxyl group of SA.

The radial distribution function can be used to describe the interaction between atoms and the distribution of chemical bonds. As shown in Figure 5a, the curve runs from left to right, and the first peak is the original bond of the substance. The second peak was mainly analyzed, which represents the first spherical layer of the central atom that is also called the first coordination layer. Figure 5a shows the radial distribution functions of Al and O. When r was 2.6 Å, the coordination number of the first coordination layer began to increase. The adsorption distance between most O atoms in water molecules and the Al in FA was 2.6 Å, which was less than the sum of the van der Waals radii of O (1.4 Å) and Al (2.5 Å), indicating a van der Waals force between the water molecules and the surface of the FA. Furthermore, the distance was far less than the sum of the van der Waals radii of O and Al, suggesting the formation of new chemical bonds between O and Al. Similarly, Figure 5b shows that the coordination number of Fe and O increased in both the first (2.6 Å) and the second (3.8 Å) coordination layers, and multiple Fe atoms may coordinate to the same O atom. The adsorption distance of the first coordination layer (2.6 Å) was much smaller than the sum of the van der Waals radii of Fe (2.2 Å) and O (1.4 Å), indicating that Fe and O formed new chemical bonds. Figure 5c shows that the radius between the atoms was greater than the sum of the van der Waals radii of Si (2.1 Å) and O (1.4 Å) in the first coordination layer (r = 3.8 Å), with a lower peak value corresponding to weak adsorption of Si on O atoms, which may be physical adsorption without the formation of chemical bonds. It is preliminarily determined that the adsorption of water molecules by FA involved both chemical and physical adsorption.

#### 2.3.4. Mean Square Displacement

Figure 5d shows the MSD of water molecules in FA at different temperatures. The order of the slope from largest to smallest is 358 K, 318 K, 338 K, and 298 K, with diffusion coefficient D values of 7.61 × 10^−6^ cm^2^/s, 2.57 × 10^−5^ cm^2^/s, 2.41 × 10^−5^ cm^2^/s, and 2.52 × 10^−5^ cm^2^/s, respectively. The high temperature accelerated the thermal motion of water molecules, allowing water molecules to diffuse faster to the FA surface. As shown in Figure 5e, the MSD slopes of water molecules in S-FA remained basically unchanged at different temperatures. Therefore, the wettability of the FA surface can be controlled using a certain range of heating methods, but this cannot affect the wettability of S-FA.

#### 2.3.5. SA Bond Angle and Carboxyl Trajectory

Due to the electrostatic interaction between the nonpolar long alkyl chains of SA, the change in the long-chain angle of SA with time was explored. As shown in Figure 5f,g, as the simulation time increased from 2 ps to 8 ps, the angle of the SA gradually decreased from 175.9° to 107.6° and finally tended to form a closed loop. In addition, a change in the long-chain angle caused the intramolecular tension to increase [26], generating a steric hindrance effect [27]. Such a spatial effect may have a repulsive effect on the proximity of water molecules, and it also affects the attack of a carboxyl group (-COOH) on the hydroxyl group (-OH) on the surface of minerals. Therefore, an excessive amount of SA led to a decrease in the grafting rate and poor surface modification. As shown in Figure 5h,i, the carboxyl group of SA moved toward the hydroxyl group, and the trajectory stacked near (-OH), which indicates that the action site of -COOH was (-OH) on the surface of FA and stayed around the hydroxyl group for a long time. Therefore, the modification of FA by SA involved a chemical reaction of (-COOH) attack (-OH).

#### 2.3.6. Hydrogen Bonds

**Figure 6:**
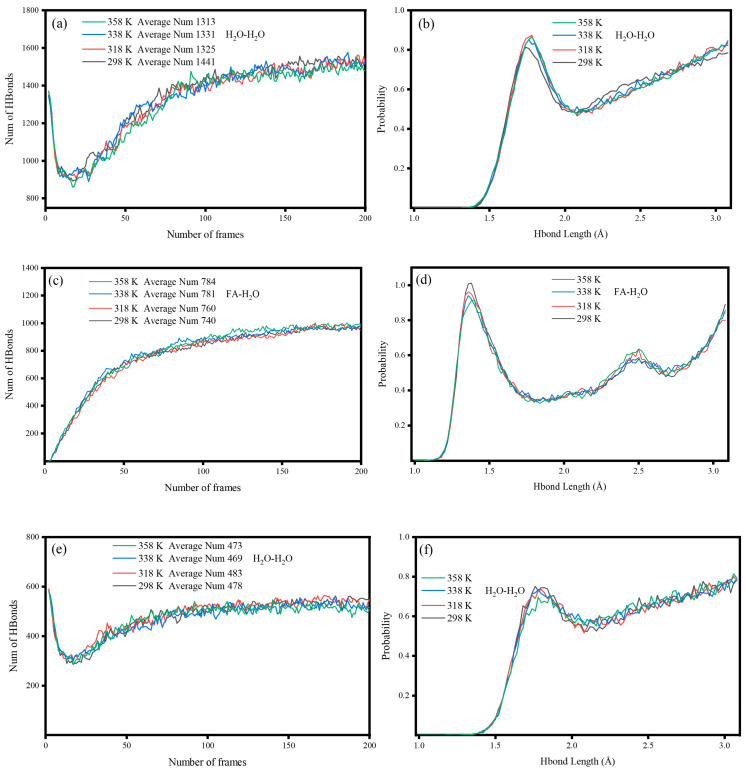

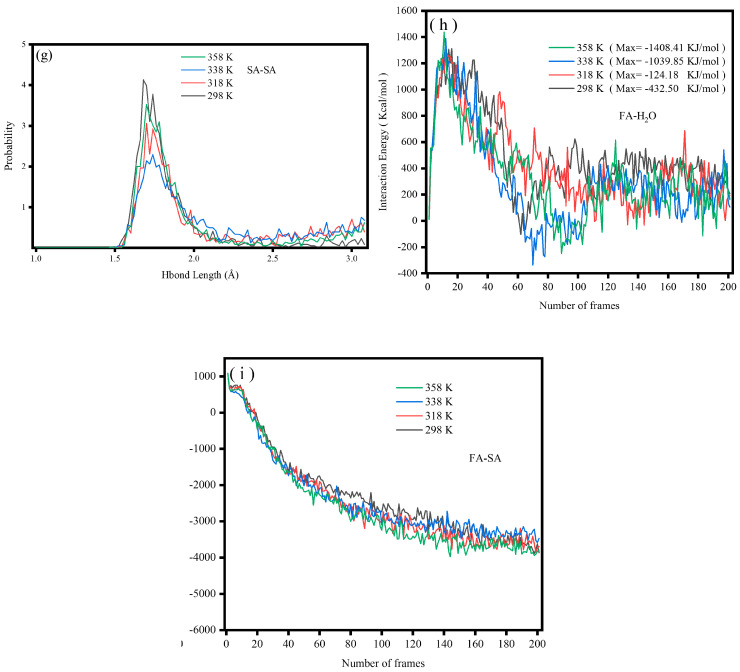
Several hydrogen bonds: (**a**,**b**) H_2_O−H_2_O of FA; (**c**,**d**) FA−H_2_O of FA; (**e**,**f**) H_2_O-H_2_O of S−FA; (**g**) SA-SA of S-FA; (**h**) interaction energy of FA-H_2_O in FA; (**i**) interaction energy of FA-SA in S−FA.

Figure 6a,c,e show the number of hydrogen bonds at 298 K, 318 K, 338 K, and 358 K, respectively. In the temperature range of 298–358 K, there was little effect on the formation of hydrogen bonds in the FA-H_2_O system. Comparing Figure 6a,e, S-FA inhibited the formation of hydrogen bonds between water molecules and water molecules in the nearby water molecular layer, which reduced the boiling point and viscosity of the whole system. Figure 6d shows the distribution of the hydrogen bond length between the FA surface and water molecules. The adsorption of water molecules on the FA surface resulted in the formation of hydrogen bonds with lengths ranging from 1.5 to 2.5 Å. The corresponding hydrogen bond became stronger with decreasing bond length, increasing the viscosity of the FA surface [28], which made it easier for FA to agglomerate in the water system. The agglomerates played a role in coating water molecules, which led to a further increase in water absorption. Figure 6g shows the distribution of hydrogen bonds between and within SA molecules. At 338 K, the distribution of hydrogen bonds with a bond length of 1.5–2.5 Å decreased, and the formation of intramolecular hydrogen bonds of SA affected the grafting of SA molecules on the surface of FA [29]. As shown in Figure 5g, intramolecular hydrogen bonds were formed after SA was closed, and this closed-loop effect may hide the carboxyl group (-COOH). Therefore, 338 K was considered as the optimal temperature for SA grafting on the surface of FA.

#### 2.3.7. Interaction Energy

Figure 6h shows the change in the interaction energy between FA and water with time. The interaction energy was less than 40 kJ/mol at 298–358 K, and the maximum reached was −1408.41 kJ/mol, demonstrating that FA chemically adsorbed with water at room temperature at 298 K [30], which is consistent with the conclusions of Section 2.3.3. Moreover, with increasing temperature, the interaction energy between FA and water molecules increased, which is related to the stronger interaction effect between FA and water. As shown in Figure 6i, the negative value of the interaction energy between FA and SA increased with increasing temperature, which indicates that the binding of FA and SA increased with increasing temperature in the temperature range of 298 K to 358 K, which implies that high-temperature conditions promoted the surface modification of FA by SA. According to the conclusions in Section 2.3.3, the main reason for the constant wettability of S-FA is that the hydrophobic layer of SA can exist stably within this temperature range, which can effectively isolate the interaction between water molecules and the surface of FA and ensure that the wettability of S-FA surface is not affected by temperature. 

#### 2.3.8. Density of States

The density of states (DOS) can be employed to study the spatial distribution of electrons in a material structure. In addition, the projection of the density of states onto the orbital, called the partial density of states (PDOS), can be used for atomic orbital analysis.

The results in Section 2.3.3 show that new chemical bonds may form between atoms closer to each other. Therefore, the partial density of states of several adsorption configurations was calculated. According to the modern valence bond theory [31], the essence of covalent bonds is that orbital overlap occurs when atoms are close to each other, and atoms bond by sharing electron pairs with opposite spins to reduce energy.

**Figure 7:**
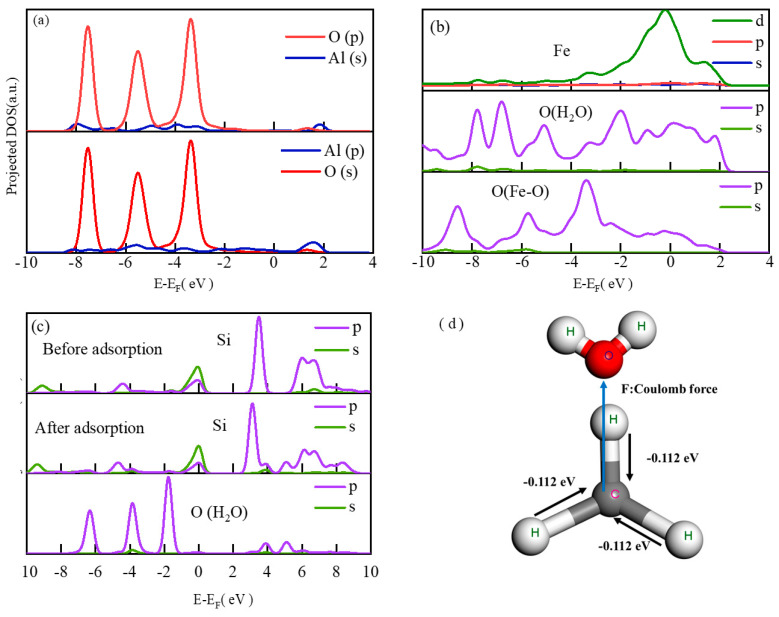
DFT calculations: (**a**–**c**) density of states plot; (**d**) Mulliken analysis.

As shown in Figure 7a, the partial density of states of the Al and O atoms in water was in the low-energy region of −8 eV to −2 eV, and the p orbital and s orbital of the Al atom overlapped with the p orbital of the O atom. The p-orbital and s-orbital of the Al atom possessed a greater degree of overlap in the number of electronic states near −8 eV and −4 eV. The partial hybridization of the s and p orbitals of the Al atom overlapped with the p orbital of the O atom to form a new chemical bond, which indicates that the top adsorption of water molecules by the Al atom on the FA surface was strong chemical adsorption [32]. This indicates that Al and Fe will preferentially undergo chemisorption with water molecules and form a more stable complex state. Al, Fe, and Si have a competitive adsorption relationship when adsorbing water molecules, and the Si atom is the main hydrating active atom of various types of concrete. Such weak competition may lead to Si being at a disadvantage in hydration. This results in a slower hydration rate and low hydration degree, which affects the final performance of the product.

As shown in Figure 7b, the low-energy region from −10 eV to 0 eV and the high-energy region from 0 eV to 2 eV were mainly contributed by the d orbital of the Fe atom. The O atom directly connected to the Fe atom was dominated by the p-orbital contribution in the entire energy level region, its p-orbital overlap region with the O atom in the water molecule was larger than that of the Fe atom, and the overlapping orbital energy was lower. The adsorption of water molecules on the surface of FA mainly depended on the p orbital interaction between O atoms, and there was a small overlap between the d orbital of the Fe atom and the p orbital of the O atom in the water molecule. Therefore, the d orbital of the Fe atom and the p orbital of the O atom directly connected to the Fe interacted with the p orbital of the O atom in the water molecule to form a new chemical bond. The two Fe atoms were paired with the O atom in the same water molecule, achieving the adsorption of water molecules on the FA surface, which was consistent with the conclusion of geometric determination in RDF.

As shown in Figure 7c, the Si atoms on the FA surface had more p-orbital electron states in the high energy level region from 0 eV to 8 eV, indicating that the number of free electrons in the p-orbital of the energy level region was greater, and the reactivity was better. However, after interacting with water molecules, the electron density of the free state almost did not decrease, the electron density of the bound state did not increase significantly, and the number of electronic states of Si atoms and O atoms in water molecules in the low-energy region almost did not overlap, which proves that the adsorption of water molecules by Si atoms on the surface of quartz is physical adsorption without new chemical bonds.

#### 2.3.9. Mulliken Charge Distribution Analysis

As shown in Table 2 and Figure 7d, the Mulliken atomic charge was calculated after the interaction between the alkyl long chain and water. When the water molecule was close to the alkyl long chain, the central C atom in the (-CH_3_) structure obtained electrons from the three H atoms in the space, increased its electronegativity, and increased the Coulomb force (repulsion) with the O atom in the water molecule. The H atom in the (-CH_3_) structure lost electrons, and the electropositivity increased, but the main Coulomb force was only one H atom in one direction. The H atom increased the Coulomb force (attraction) with the O atom in the water. At the same time, the Coulomb force (repulsive force) of the H atom in water also increased. Therefore, the Coulomb force (repulsive force) of the (-CH_3_) structure to the water molecule generally increased, which played a role in eliminating the water molecule [33]. This promoted the stable existence of long alkyl chains at water interfaces.

## 3. Materials and Methods

### 3.1. Materials

The FA selected for the experiment was obtained from Bijie Qianxi Xingqian Building Materials Co., Ltd. (Qianxi, Guizhou, China). Coal gasification fly ash is produced during coal gasification. The combustion loss of coal gasification fly ash at 150–1000 °C is 8.619%. Gasification fuel is lignite, gasification technology: Space pulverized coal pressurized gasification technology (HT-L) is a coal gasification technology with independent intellectual property rights developed by Aerospace Long March Chemical Engineering Co., Ltd. (Beijing, China), China Academy of Launch Vehicle Technology (Beijing, China) (gasification temperature is generally controlled between 1300 and 1750 °C and adjusted according to the ash melting point and viscose-temperature characteristics of coal; gasification pressure is 4.0–4.2 Mpa). The selected surfactant SA was provided by Indonesian SA 1801 (Dongguan, China).

### 3.2. Modification Methods

FA was modified using the dry method according to the SA accounting for 1.2–4% of the FA mass [34]. Certain amounts of FA and SA were placed in a beaker and stirred for 15 min at a stirring rate of 1000 r/min in a constant-temperature water bath at 70 °C.

### 3.3. Oil Absorption Value

The mass of the beaker and the glass rod were weighed. Approximately 5 g of the sample was weighed and poured into a beaker. Linseed oil was added dropwise and fully stirred with a glass rod. When the powder formed an agglomerate, the reaction was stopped, and the total mass was determined. The oil absorption value was calculated according to Equation (1).

(1) $$D=\frac{m_{3}-m_{2}}{m_{2}-m_{1}}$$

where *m*_1_ is the quality of the beaker and glass rod, *m*_2_ is the mass of the beaker and glass rod + FA, and *m*_3_ is the quantity of the beaker, glass rod, FA, and flax oil.

### 3.4. Activation Index

Approximately 5 g of the sample was weighed and placed in a 250 mL beaker containing 150 mL of distilled water. The mixture was shaken for 1 min at a rate of 120 times per minute and allowed to stand at room temperature for 45 min. After obvious stratification, the floating matter was removed from the water, filtered to obtain the material at the bottom of the beaker, dried in a hot air oven at 80 °C until constant weight, and weighed. The activation index was calculated according to Equation (2):

(2) $$K=1-\frac{D}{M}\times 100\%$$

where *D* denotes the quality of sediment, and *M* indicates the total mass of the sample.

### 3.5. Characterization Method

The phase identification of the samples was carried out using X-ray powder diffraction (XRD, Rigaku Ultima IV, Tokyo, Japan). The test conditions included a Cu emission source, a scanning range of 10–80°, and a scanning speed of 10°/min for continuous scanning. The atlas results were analyzed with HighScore Plus software 3.0.5.15611.

The morphology of the samples was observed with a 3.0 kV scanning electron microscope (SEM, ZEISS Gemini SEM 300, Oberkochen, Germany).

The chemical groups of the samples were studied using Fourier transform infrared (FT-IR) spectroscopy (Thermo Fisher Scientific Nicolet iS20, Waltham, MA, USA) with a range of 400–4000 cm^−1^ using the KBr tableting method.

The specific surface area and pore volume of the samples were analyzed via an automatic specific surface area porosity analyzer (BET, Micromeritics ASAP 2460, Shanghai, China) under a nitrogen atmosphere.

Thermogravimetric analysis (TG, Netzsch TG 209 F1, Shanghai, China) was used to determine the amount of drug coating on the surface of the sample with a temperature range of 25–1000 °C at a heating rate of 15 °C/min.

The hydrophobicity of the sample surface was investigated using a contact angle/surface tension meter (CA, SDC 350KS, Guangzhou, China).

### 3.6. Coverage and Coverage Test

The specific surface area of FA was measured using an automatic specific surface area porosity analyzer. The percentage of mass loss of FA and S-FA in the range of 200–300 °C was obtained from TG curves. The coating amount (mg/g) was calculated using Formula (3), the coating rate (%) was determined from Formula (4), and the SA utilization rate (%) was calculated using Formula (5):

(3) $$q=\left(B-A\right)\times 100$$

(4) $$p=\frac{q\times M}{S}\times 100\%$$

(5) $$Z=\frac{q\times C}{D}\times 100\%$$

where *q* is the covering quantity, *p* is the coating rate, *Z* is the utilization rate of SA, *S* is the specific surface area of FA, *C* is the quality of S-FA, *D* is the amount of SA, *A* is the mass loss percentage of FA (200–300 °C), *B* is the mass loss percentage of S-FA (200–300 °C), and *M* is the cross-sectional area of 1 mg of SA (0.4655 m^2^).

### 3.7. Model Building

#### 3.7.1. Construction of the Initial FA Model

The XRD data of FA were analyzed using HighScore Plus software, and the first seven main minerals with the highest content were selected to establish the initial cell model for each mineral. In Materials Studio 19.1.0.1740 (MS) software, the initial unit cell model was transformed into a nonperiodic structure, and the amorphous cell (AC) module was used to mix the mineral content in the refined results to establish an amorphous model of FA with different densities. Then, the simulated XRD data of the amorphous model with the experimental XRD data were compared using the Reflex Refinement module, and the variance of the graph fitting was calculated. When Rwp [35] and Rp were less than 15%, the XRD main peak of the simulated structure of the FA was consistent with the XRD experimental value of the FA for the amorphous structure. This indicates that the simulated structure is close to the real structure.

#### 3.7.2. Calculation Method

Three models were calculated, including the interaction between FA and the water molecular layer, the interaction between FA and the SA layer, and the interaction between S-FA and the water molecular layer after the surface of FA was covered with the SA layer. To eliminate the periodic boundary effect, a 30 Å vacuum layer was added above the water molecular layer.

Using the Forcite module of MS software, the structure of the initial model was optimized via the steepest descent method. In this work, the COMPASS II force field was selected and applied to all simulation processes. The force field was extensively verified to accurately describe SA, the FA surface, and their interaction characteristics. The density of the simulated system was adjusted to match the density of S-FA under experimental conditions. This was undertaken by changing the size of the simulation box or adjusting the number of molecules in the system. After each density adjustment, the simulated XRD pattern was compared with the experimental XRD pattern, and the graph fitting variance was calculated to ensure that the simulated parameters approximated the experimental parameters. Since the model obtained by the AC module is in an energy-unstable state, the model needs to be annealed (Anneal) [36]. A stable structure of the system was obtained by calculating the cycle process of heating and cooling the system. The selection of annealing temperature parameters is based on the fact that the annealed model can achieve energy and structural stability, and there are no energy anomalies and bond length and bond angle anomalies. The initial annealing temperature ranged from 300–500 K, and five cycles were performed. The molecular dynamics relaxation at different temperatures was performed under the NVT canonical ensemble [37]. The time step was set to 1 fs, and the total time was 20 ps. The radial distribution function, mean square displacement, hydrogen bond number, and interaction energy were obtained via relaxation. According to the relevant MD results, some structures were selected for DFT calculations. DFT calculations were performed using the Castep module in MS software. The generalized gradient approximation [38] and the Perdew Burke Ernzerhof (PBE) functional were used to address the exchange-related potentials, and the density of states and Mulliken charge layout analysis were calculated.

## 4. Conclusions

(1) FA was changed from a hydrophilic structure to a hydrophobic structure through the surface modification of SA. When the activation index increased from 0 to 0.98, the contact angle increased from 23.4° to 127.2°, and the oil absorption decreased from 0.564 g/g to 0.510 g/g.
(2) The adsorption effect of FA on SA was mainly pore adsorption, and the pore-blocking effect was preferentially completed to start the surface coating outside the pores. In addition, the pore-blocking effect and the adhesion between particles caused by SA modification reduced the BET-specific surface area from 13.973 m^2^/g to 3.218 m^2^/g, which weakened the adsorption performance of FA.
(3) In the temperature range of 298–358 K, the surface wettability of FA became more hydrophilic with increasing temperature, while the surface wettability of S-FA was not affected in this temperature range. When there was an excess of SA, a multilayer unstable interface formed on the surface of FA, which was less stable in water than the monolayer interface formed by SA, thus reducing the activation index. In addition, when the temperature was greater than 210 °C, the SA layer began to decompose and volatilize, and the SA coating rate on the FA surface was indirectly adjusted by adjusting the reaction time and temperature to dynamically regulate the surface hydrophobicity.
(4) Al, Fe, and Si served as the main active adsorption sites of FA on H_2_O. The top adsorption of the Al atom on water molecules was strong chemical adsorption, with new chemical bonds between Al and O as well as Fe and O. The adsorption of Si atoms on water molecules was physical adsorption. In addition, the adsorbed water molecules on the surface of FA formed hydrogen bonds with lengths of 1.5–2.5 Å, which resulted in the aggregation of FA in water.
(5) In the temperature range of 298–358 K, heating increased the interaction energy between FA and SA, and the combination of FA and SA became more stable with increasing temperature. However, according to the intramolecular hydrogen bond length of SA, 338 K was considered as the best temperature for SA grafting on the FA surface.
(6) The long alkyl chain in the hydrophobic layer of SA mainly relied on the central carbon atom in the -CH3′ structure to obtain electrons from the H atoms in different directions in the space, increasing the Coulomb repulsion with the O atoms in the water molecule to achieve the hydrophobic effect. In addition, the carboxyl group on the long chain of SA tended to attack the hydroxyl group on the surface of FA, but the interchain interactions of SA molecules led to steric hindrance and closed-loop effects. When there was an excess of SA, the carboxyl group (-COOH) in SA was hindered from attacking the hydroxyl group (-OH) on the surface of FA, resulting in the instability of the formed hydrophobic layer.

## Figures and Tables

**Table 1:** R-weighted pattern and R-pattern.

Density (g/cm^3^)	1.0	1.2	1.4
Rwp (%)	6.55	6.24	6.95
Rp (%)	3.43	3.66	4.08

**Table 2:** Mulliken analysis.

Atom	Before Interaction	After Interaction
H(-CH_3_)	0.053	0.165
C(-CH_3_)	−0.159	−0.488

## Data Availability

All data that support the findings of this study are included within the article.
